# Risk of Death From Pulmonary Tuberculosis Attributable to Diabetes Mellitus in Brazil: A Retrospective Cohort Study of Health Surveillance Data

**DOI:** 10.7759/cureus.103262

**Published:** 2026-02-09

**Authors:** Maria E Nadaf, Elisabeth C Duarte, Cor J Fontes

**Affiliations:** 1 Department of Infectious Diseases, Hospital Universitário Júlio Müller, Cuiabá, BRA; 2 Tropical Medicine Unit, Universidade de Brasília, Brasília, BRA; 3 Faculty of Medicine, Universidade Federal de Mato Grosso, Cuiabá, BRA

**Keywords:** attributable risk, diabetes, fatality rate, tuberculosis, tuberculosis-diabetes comorbidity

## Abstract

Background: Diabetes mellitus (DM) is associated with increased susceptibility to tuberculosis (TB) and unfavorable treatment outcomes.

Objective: To evaluate the risk of pulmonary tuberculosis (PT)-related mortality attributable to DM in Brazil and to identify subgroups that may require differentiated clinical management.

Methods: Descriptive and retrospective cohort study using data from Brazil’s national health surveillance system. Data on new PT cases in individuals aged ≥18 years, diagnosed between 2012 and 2017, were analyzed. Fatality rates were compared between patients with and without self-reported DM, stratified by relevant covariates, to estimate the attributable risk due to DM (ARDM%).

Results: A total of 355,659 patients with PT were included in the analysis of this study, of whom 29,579 (8.3%) had PT-DM, and 326,080 (91.7%) had PT alone, without DM. Patients with PT-DM exhibited higher PT fatality rates (1,691, 5.7%) and lower treatment discontinuation rates (2,425, 8.2%) than those without DM (12,203, 3.7%) or who completed the full course of treatment (50,216, 15.4%). Overall, DM accounted for a 34.6% increase in PT fatality (ARDM%). The highest ARDM% (>50%) was observed among patients with higher education levels, those aged 18-39 years, residents of peri-urban areas, recipients of the Brazilian Cash Transfer Program (BCTP), and incarcerated individuals.

Conclusions: DM substantially increases the fatality rate among patients with PT in Brazil, particularly in specific subgroups that may benefit from targeted clinical and public health interventions.

## Introduction

Tuberculosis (TB) is a preventable and usually curable disease. More than 10 million people continue to fall ill with TB every year. It remains a significant public health challenge in Brazil, one of the 30 countries with the highest TB burden globally [1]. From exposure to Mycobacterium tuberculosis to the progression to pulmonary tuberculosis (PT), both exogenous and endogenous factors are involved in PT progression. Among these factors, bacillary load, immunosuppression, age, malnutrition, alcohol consumption, and use of tobacco and drugs have been underscored [2]. Patients with comorbidities generally had lower treatment success rates and higher PT fatality rates [3]. Evidences indicate that diabetes mellitus (DM) is associated with increased susceptibility to infection, disease reactivation, and unfavorable outcomes of PT treatment [2-9]. The mechanisms by which DM worsens the prognosis of patients with PT are not fully understood. DM impairs the activation and function of monocytes, macrophages, and lymphocytes, which are essential host cells for the immune response against the bacillus [10,11]. Furthermore, DM contributes to treatment failure against M. tuberculosis and increases the risk of death in patients undergoing PT [10,11].

In a collective context and under conditions of endemic PT, demographic changes and population aging increase the likelihood of a high number of PT cases coexisting with DM. This dual disease burden renders strategies to address these health problems even more challenging, including delayed diagnosis, polypharmacy, drug interactions, worse prognosis, and a higher risk of death [10]. Therefore, studies have emphasized the importance of implementing effective prevention and control actions for the concurrent management of DM and PT endemics in different regions [6,12]. This study aimed to estimate the risk of death from PT attributable to DM in subgroups of PT with concomitant DM for whom a differentiated clinical management approach is warranted.

## Materials and methods

Study design and setting 

This cohort study was based on PT data recorded in the Brazilian Notifiable Diseases Information System for Tuberculosis (SINAN-TB) [13]. TB is a notifiable disease across the entire national territory. The SINAN-TB database comprises sociodemographic, risk factor, and clinical information, as well as follow-up data through the end of treatment or outcome ascertainment (death or treatment discontinuation) for all PT cases.

Study population

The study population comprised all new PT cases in individuals aged 18 years or older diagnosed between 2012 and 2017. Patients with unknown or missing outcomes or those transferred to another health service were excluded.

Data collection

The variables of interest included PT treatment outcomes - cure, death, treatment discontinuation, multidrug-resistant TB (MDR-TB), therapeutic regimen change, and therapeutic failure. Self-reported diabetes mellitus (DM) was used to classify cases as PT with DM (PT-DM) or PT without DM (PT non-DM). Additional variables included sex, skin color, education level, age, region and area of residence, HIV status and treatment, alcohol consumption, illicit substance use, tobacco use, and TB bacteriological results. Variables related to vulnerable populations - such as incarceration, homelessness, and participation in the Brazilian Cash Transfer Program (BCTP) - were included starting in 2015, when this information began to be systematically recorded in the SINAN database. HIV-positive patients were classified according to antiretroviral therapy, including those who were treated, untreated, or had unknown treatment status. For all study variables, missing data were treated as a separate analytical category and were presented in the Results section.

Statistical analysis

PT treatment outcomes were stratified according to the presence or absence of DM to assess DM and to estimate DM-attributable risks (ARs). Comparisons of fatality rates between groups defined by covariates of interest were used to estimate the PT fatality rate ratio (relative risk (RR)), absolute AR, and the AR percentage among those exposed to DM (ARDM%). All statistical analyses were performed using Stata, version 12.0 (StataCorp, College Station, TX).

Ethical considerations

This study used exclusively public, anonymized TB surveillance data. Access to the SINAN database was formally requested from the Health Surveillance Secretariat of the Brazilian Ministry of Health via the Citizen Information Service Electronic System, and the dataset was provided in February 2019. Ethical approval was granted by the Faculty of Biomedical Sciences (FACIMED) Research Ethics Committee (No. 4220431, August 18, 2020). The study followed EQUATOR Network guidelines, specifically the STROBE Extension for RECORD, throughout the research process. The data that support the findings of this study are available from the corresponding author upon reasonable request.

## Results

**Figure 1:**
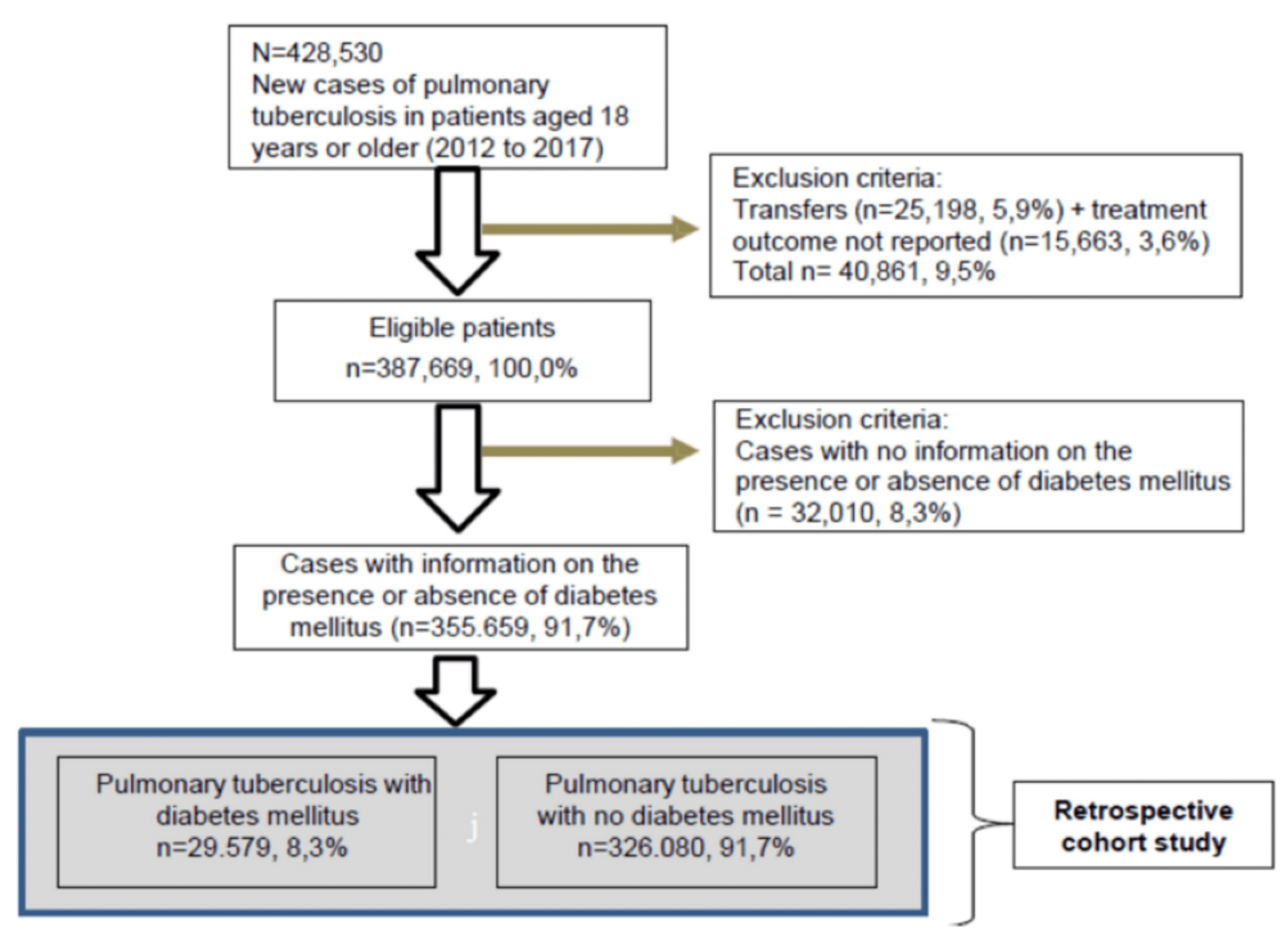
Flowchart of patients aged 18 years and older with pulmonary tuberculosis and self-reported diabetes mellitus in Brazil, 2012-2017. Image credit: All authors.

The study identified 428,530 new PT cases, of which 355,659 (83.0%) had information on both treatment outcomes and DM status. Among these, 29,579 (8.3%) were diagnosed with DM (Figure 1).

The overall PT fatality rate in the eligible study population (387,669) was (16,341, 4.2%), regardless of DM status. Based on point estimates, higher fatality rates were observed among individuals with unknown skin color (1,716, 5.7%), no schooling (1,421, 7.1%) or unknown education level (6,437, 6.5%), and among those aged over 39 years, showing a progressive increase with age - particularly in individuals aged 60-79 years (4,592, 9.2%) and those aged 80 years or older (1,152, 17.2%). Higher fatality rates were also observed among individuals experiencing homelessness (756, 7.5%), those reporting alcohol use (4,746, 6.5%), and those with unknown information for this variable (2,320, 7.8%) or for HIV status and treatment (6,698, 7.6%). Notably, the fatality rate was lower among incarcerated individuals (255, 0.9%) than among non-incarcerated individuals (9,088, 4.2%). Among HIV-positive patients receiving highly active antiretroviral therapy (HAART), the PT fatality rate (256, 2.6%) was comparable to that of HIV-negative individuals (7,789, 3.0%). In contrast, HIV-positive patients who were not on treatment (202, 5.0%) or whose treatment status was unknown (1,396, 5.3%) exhibited higher fatality rates compared with HIV-negative patients (Table 1).

**Table 1:** Fatality rates associated with pulmonary tuberculosis in individuals aged 18 and older, by selected variables, Brazil, 2012-2017 *Patients transferred or without outcome information were excluded. BCTP, Brazilian Cash Transfer Program; HIV, human immunodeficiency virus; HAART, highly active antiretroviral therapy

Characteristics	Category	Total cases	Death	
			n	Fatality rate (%)**
All cases	-	387,669	16,341	4.2
Sex	Female	113,856	3,909	3.4
	Male	273,783	12,430	4.5
Skin color	White + yellow	124,984	4,785	3.8
	Black	51,737	2,186	4.2
	Brown	177,064	7,511	4.2
	Indigenous	3,656	143	3.9
	Not informed	30,228	1,716	5.7
School level	None	20,029	1,421	7.1
	Elementary school	171,690	6,666	3.9
	High school	77,987	1,530	2
	Higher education	18,911	287	1.5
	Not informed	99,052	6,437	6.5
Age (years)	18-39	198,221	3,812	1.9
	40-59	132,539	6,752	5.1
	60-79	50,101	4,592	9.2
	80 or higher	6,705	1,152	17.2
Region of residence	Central West	17,951	678	3.8
	Southern	49,382	1,845	3.7
	South East	181,423	7,821	4.3
	North East	98,797	4,868	4.9
	Northern	40,050	1,123	2.8
Area of residence	Urban	250,193	11,156	4.5
	Rural	27,135	1,082	4
	Peri-urban	2,871	101	3.5
	Not informed	107,470	4,002	3.7
BCTP recipients	No	108,198	3,724	3.4
	Yes	9,755	353	3.6
	Not informed	269,716	12,264	4.6
Homeless person	No	231,674	8,573	3.7
	Yes	10,099	756	7.5
	Not informed	145,896	7,012	4.8
Incarcerated people	No	213,683	9,088	4.2
	Yes	29,288	255	0.9
	Not informed	144,698	6,998	4.8
Status of HIV and HIV treatment	HIV-negative	259,674	7,789	3
	HIV-positive (total)	40,477	1,854	4.6
	HIV-positive on HAART	9,963	256	2.6
	HIV-positive without HAART	4,014	202	5
	HIV-positive and not informed HAART	26,500	1,396	5.3
	Not informed both HIV and HAART	87,518	6,698	7.6
Alcohol consumption	No	284,970	9,275	3.2
	Yes	73,099	4,746	6.5
	Not informed	29,600	2,320	7.8
Illicit substance use	No	202,815	7,679	3.8
	Yes	38,525	1,272	3.3
	Not informed	146,329	7,390	5
Tobacco use	No	189,050	6,632	3.5
	Yes	52,990	2,505	4.7
	Not informed	145,629	7,204	5
Bacilloscopy/Culture result	Negative	14,613	365	2.5
	Positive bacilloscopy or culture	208,698	7,292	3.5
	Positive both, bacilloscopy and culture	59,014	1,658	2.8
	Not informed	105,344	7,026	6.7

Table 2 presents the treatment outcomes of PT among patients with and without DM (355,659). In this population, the overall cure rate was 267,425 (75.2%), and the PT-related fatality rate was 13,894 (3.9%). Treatment discontinuation and death from causes other than PT accounted for 52,641 (14.8%) and 15,950 (4.5%) of cases, respectively. The prevalence of DM among patients with PT was 29,579 (8.3%; 95% CI, 8.2-8.4%). Compared with patients without DM, those with PT-DM had higher fatality rates due to PT (1,691, 5.7% vs. 12,203, 3.7%; RR = 1.53) and due to other causes (1,563, 5.3% vs. 14,387, 4.4%; RR = 1.20). However, patients with PT-DM also had higher cure rates (23,300, 78.8% vs. 244,125, 74.9%; RR = 1.05) and substantially lower treatment discontinuation rates (2,425, 8.2% vs. 50,216, 15.4%; RR = 0.53).

**Table 2:** Distribution of pulmonary tuberculosis (PT) cases by outcome and presence of diabetes mellitus (DM), 2012-2017, Brazil. *Persons who were transferred or lacked outcome information, as well as those who did not report DM status, were excluded. **Proportion of PT cases with known outcomes (cure or death) relative to the total number of cases (355,659). ^a^Prevalence of DM among PT cases relative to the total number of PT cases with reported DM status. ^b^Prevalence of DM among PT cases relative to the total number of PT cases with reported DM status and known outcomes (cure or death). MDR-TB, multidrug-resistant pulmonary tuberculosis

Outcomes	Total		PT-non-DM		PT-DM		RR
	n	%	n	%	n	%	
Outcome status (total *N* = 355,659*)							
Cure	267,425	75.2	244,125	74.9	23,300	78.8	1.05
Death from PT	13.894	3.9	12,203	3.7	1,691	5.7	1.53
Death from other causes	15.950	4.5	14,387	4.4	1,563	5.3	1.2
Treatment discontinuation	52.641	14.8	50,216	15.4	2,425	8.2	0.53
MDR-TB	4,359	12.2	3,903	1.2	456	1.5	1.28
Drug regimen changed	1,149	0.3	1,026	0.3	123	0.4	1.35
Therapeutic failure	241	0.1	220	0.1	21	0.1	1
Total	355,659	100	326,080	100	29,579	100	-
DM prevalence (95% CI): 8.3% (8.2%-8.4%)^a^							
Outcome status for cure/death (*n* = 297.269, 83.6%**)							
Cure	267425	89.4	244,125	90.2	23,300	87.8	0.97
Death from PT	13894	5	12,203	4.5	1,691	6.4	1.41
Death from other causes	15950	5.5	14,387	5.3	1,563	5.9	1.11
Total	297269	100	270,715	100	26,554	100	-
DM prevalence (95% CI) among cases with known outcome: 8.9% (8.8%-9.0%)^b^							

Because patients with PT-DM are less likely to discontinue treatment - that is, more likely to have recorded outcomes (cure or death) - this could lead to overestimation of PT treatment outcomes compared with patients with PT non-DM. Therefore, comparisons were restricted to cases with a known treatment outcome status (cured or deceased), representing 83.6% of all cases (355,659). Among these, 26,554 (8.9%; 95% CI: 8.8-8.9%) patients had PT-DM. In this comparison, patients with PT-DM exhibited poorer outcomes over the follow-up period than those without DM, with a slightly lower cure rate (23,300, 87.8% vs. 244,125, 90.2%; RR = 0.97) and higher fatality rates due to PT (1,691, 6.4% vs. 12,203, 4.5%; RR = 1.41) and due to other causes (1,563, 5.9% vs. 14,387, 5.3%; RR = 1.11) (Table 2).

Patients with PT-DM had a 53% higher fatality rate than PT non-DM patients (RR = 1.53), corresponding to an absolute excess of 1.98 deaths per 100 patients. This represents a 34.61% increase in the fatality rate attributable to DM among patients with PT-DM (ARDM%) (Table 2).

The ARDM% was similar between women (38.38%) and men (34.83%) but was more pronounced among Black individuals (40.22%), those with higher education levels (high school: 54.23%; higher education: 53.40%), individuals aged 18-39 years (50.43%), residents of the Southeastern region (40.69%), those living in peri-urban areas (66.98%), BCTP recipients (53.86%), individuals not experiencing homelessness (41.87%), and incarcerated individuals (80.92%). Regarding HIV status, DM had a greater impact on PT fatality rates (ARDM%) among HIV-positive individuals receiving HAART (36.11%) and among HIV-negative individuals (35.57%). Among patients reporting alcohol, drug, or tobacco use, DM was associated with increased PT fatality rates, with ARDM% values ranging from 39.23% to 49.59% across these subgroups, with ARDM% values ranging from 39.23% to 49.59% across these subgroups. Across the different sputum test results (smear microscopy or culture), DM exerted a substantial yet similar influence on PT fatality rates, with ARDM% values ranging from 31.85% to 40.64% across these categories (Table 3). Interestingly, PT-DM appeared to be a protective factor in certain subgroups, including individuals aged 60-79 years, Indigenous people, and residents of the Central-West region, with reductions in the risk of death attributable to DM (ARDM%) of 22.39%, 8.91%, and 3.60%, respectively (Table 3).

**Table 3:** Fatality rates and measures of association between the risk of death and self-reported diabetes mellitus (DM) among adults with pulmonary tuberculosis (PT), according to selected variables, 2012-2017, Brazil (N = 355,659*). *Persons transferred or without outcome information, as well as those who did not report DM status, were excluded. AR(DM) = Absolute risk difference for DM among the exposed, where *exposed* are pulmonary PT cases with DM (PT-DM): expresses the absolute risk difference of PT death between people with and without DM. FR, fatality rate; RR, relative risk; HIV, human immunodeficiency virus; BCTP, Brazilian Cash Transfer Program; HAART, highly active antiretroviral therapy

Variable	Category	Death	(PT non-DM)	Death	(PT-DM)	RR	Attributable risk	
		*n* = 326,080		*n *= 29,579				
		Fatality rate		Fatality rate		(a/b)	AR(DM)	AR(DM)%
		n	% (a)	n	% (b)		(b-a)	((b-a)/b)
Total	-	12,203	3.7	1,691	5.7	1.53	1.98	34.61
Sex	Female	2,811	3.0	521	4.8	1.62	1.85	38.38
	Male	9,391	4.1	1,170	6.2	1.53	2.17	34.83
Skin color	White + yellow	3,773	3.5	530	5.1	1.46	1.61	31.38
	Black	1,643	3.8	219	6.3	1.67	2.53	40.22
	Brown	5,522	3.7	783	5.8	1.55	2.04	35.42
	Indigenous	126	3.9	6	3.6	0.92	-0.32	-8.91
	Not informed	1,139	4.8	153	7.6	1.57	2.76	36.32
School level	None	1,089	6.6	152	7.4	1.11	0.75	10.16
	Elementary school	5,229	3.6	720	5.2	1.45	1.6	30.89
	High school	1,201	1.7	189	3.8	2.18	2.05	54.23
	Higher education	231	1.4	41	2.9	2.14	1.57	53.40
	Not informed	4,453	5.8	589	8.2	1.42	2.4	29.41
Age (years)	18-39	3,076	1.7	149	3.5	2.02	1.76	50.43
	40-59	4,980	4.7	733	4.8	1.02	0.1	2.09
	60-79	3,277	8.9	664	7.3	0.82	-1.63	-22.39
	80 or higher	837	16.0	145	16.7	1.04	0.68	4.07
Region of residence	Central-West	504	3.4	37	3.3	0.96	-0.12	-3.60
	Southern	1,515	3.5	171	5.2	1.50	1.75	33.46
	South East	5,928	3.8	807	6.4	1.69	2.6	40.69
	North East	3,417	4.3	534	6.0	1.39	1.71	28.31
	Northern	834	2.5	142	3.8	1.50	1.27	33.24
Area of residence	Urban	7,859	3.9	1,175	5.6	1.45	1.76	31.20
	Rural	843	3.7	75	4.4	1.71	0.64	14.61
	Peri-urban	68	2.8	12	8.6	3.03	5.78	66.98
	Not informed	3,433	3.5	429	6.2	1.79	2.75	44.14
BCTP recipients	No	2,980	3.2	424	4.7	1.49	1.55	32.77
	Yes	255	3.1	69	6.7	2.17	3.63	53.86
	Not informed	8,968	4.0	1,198	6.1	1.53	2.12	34.64
Homeless person	No	6,876	3.4	1,036	5.8	1.72	2.42	41.87
	Yes	657	7.2	27	7.8	1.08	0.59	7.58
	Not informed	4,670	4.2	628	5.6	1.33	1.39	25.04
Incarcerated people	No	7,339	3.9	1,046	5.8	1.50	1.94	33.22
	Yes	200	0.8	16	3.9	5.24	3.18	80.92
	Not informed	4,664	4.2	629	5.6	1.34	1.41	25.22
HIV status and treatment	HIV-negative	6,235	2.8	912	4.3	1.55	1.54	35.57
	HIV-positive on HAART	213	2.3	11	3.6	1.56	1.30	36.11
	HIV-positive without HAART	162	4.4	6	4.5	1.03	0.12	2.64
	HIV-positive and not informed about HAART	1,074	4.6	42	5.5	1.21	0.97	17.54
	Not informed	4,519	6.8	720	9.8	1.44	3.03	30.79
Alcohol consumption	No	8,062	3.1	1,089	4.7	1.50	1.55	33.26
	Yes	3,861	6.0	461	9.9	1.65	3.90	39.23
	Not informed	280	8.6	141	8.9	1.03	0.27	3.03
Illicit substance use	No	6,587	3.6	969	5.6	1.55	1.98	35.48
	Yes	1,110	3.1	67	6.1	1.98	3.03	49.59
	Not informed	4,506	4.2	655	5.9	1.40	1.67	28.35
Bacilloscopy/Culture result	Negative	296	2.3	33	3.4	1.49	1.11	32.84
	Positive bacilloscopy or culture	5,609	3.2	784	4.7	1.47	1.5	31.85
	Positive both, bacilloscopy and culture	1,345	2.6	187	4.4	1.68	1.78	40.64
	Not informed	4,953	5.7	687	9.0	1.56	3.23	36.09

## Discussion

This study identified a DM prevalence of 29,579/355,659 (8.3%) among all new adult cases of PT. Among patients with DM, the proportion of treatment discontinuation was markedly lower (2,425, 8.2%) than among those without DM (50,216, 15.4%). In contrast, the fatality rate for PT was higher among patients with DM (1,691, 6.4%) than among those without DM (12,203, 4.5%), with 34.6% of PT-related deaths among patients with PT-DM being attributable to DM. The impact of DM on the risk of death from PT was greater among women, young adults, individuals with higher education levels, residents of the Southeastern region, those living in urban and peri-urban areas, and individuals who reported alcohol, tobacco, or illicit drug use.

The prevalence of DM among adult PT cases (29,579, 8.3%) was slightly higher than that reported by Abreu et al., who found a DM prevalence of 7.2% among individuals with PT [14]. The lower prevalence in that study may be explained by the inclusion of patients younger than 18 years [14]. The prevalence of self-reported DM observed in the present study also exceeded that reported for the overall Brazilian adult population [15], supporting the hypothesis that DM increases susceptibility to PT [2,3].

Although patients with PT-DM exhibited poorer clinical outcomes, the proportion of patients who were non-adherent to treatment was significantly lower in this group. Similar findings were reported by Silva et al., who observed lower rates of PT treatment discontinuation among individuals with PT-DM compared with those without DM. In a multivariate analysis, a protective trend of DM against treatment discontinuation was identified, although this association was not statistically significant [16]. The need for continuous contact with health services for glycemic monitoring and medication access may contribute to better compliance with PT treatment among patients with DM, supporting the findings of the present study [17,18].

The fatality rate for PT was higher among patients with DM, with DM accounting for a 34.6% increase in the risk of PT-related deaths attributable to PT-DM comorbidity. Indeed, DM has been consistently identified as a significant risk factor associated with poor TB treatment outcomes, including increased mortality from both PT and other causes of death [3,19]. In a cohort study, Dooley and Chaisson reported that PT patients with DM had 6.5-fold higher odds of death from PT than those without DM, even after adjustment for HIV status, age, weight, and place of birth [10].

A hyperglycemic state negatively affects TB treatment outcomes, creating challenges such as an increased likelihood of multidrug-resistant TB (MDR-TB), reduced bioavailability of rifampicin (an important drug used in PT treatment), and a higher risk of progression from latent TB infection to active disease [6,8,10,20,21]. Rifampicin also accelerates the metabolism of sulfonylurea hypoglycemic agents, reducing their serum concentrations and, consequently, their effectiveness [22]. Another study demonstrated that the bioavailability of rifampicin was 53% lower in patients with PT-DM than in those with PT alone and that the degree of hyperglycemia was inversely associated with rifampicin bioavailability [20].

The impact of DM on the risk of death from PT was greater among women and young adults (18-39 years). These groups had relatively low fatality rates in the absence of DM (2,811, 3.0% and 3,076, 1.7%, respectively), which increased markedly when DM was present. For example, among young adults aged ≤39 years, the PT fatality rate increased from 1.7% in the absence of DM to 149, 3.5% in its presence, corresponding to an ARDM% of 50.43%. In contrast, among older adults (≥80 years), the fatality rate for PT was already high, with minimal variation between those with and without DM. Thus, the relative impact of DM on PT-related mortality was proportionally lower in this age group.

The vulnerability of certain social groups affects not only their physical and immunological conditions but also their access to health services for timely diagnosis and treatment. This was evident in the present study, which demonstrated a high AR due to diabetes mellitus (ARDM%) among Black or mixed-race individuals and recipients of the BCTP. Conversely, the greatest contribution of DM to the increase in the PT fatality rate was observed among groups traditionally at lower risk of death from PT, such as individuals with higher education levels. In these groups, PT fatality rates were low in the absence of DM but increased markedly when DM was present. In contrast, among individuals with no formal education, DM contributed minimally to the risk of death, as their baseline PT fatality rate was already high and remained similar regardless of DM status.

Greater DM-attributable impacts on PT fatality rates were also observed among residents of the Southeastern region and those living in urban and peri-urban areas. Peri-urban areas represent transitional zones between urban and rural regions, typically located beyond the suburbs of large metropolitan centers. In Brazil, these areas are characterized by complex sociodemographic conditions and limited availability and accessibility of health services. They often include extensive favelas on the outskirts of highly developed cities. As reported by Ferreira et al., the interplay of poverty, inadequate living conditions, and restricted access to healthcare creates a precarious situation for populations residing in these peripheral areas [23]. Although the number of individuals in this category was relatively small, the present study observed that the presence of DM was significantly associated with increased risk of death from PT, underscoring the need for targeted healthcare strategies and greater attention to these vulnerable groups.

Among individuals who reported alcohol, tobacco, or illicit drug use, DM contributed to 39.23%, 48.03%, and 49.59%, respectively, of the fatality rate of PT among patients with PT-DM. These behaviors are known to negatively affect adherence to treatment regimens [24,25]. Therefore, patients with PT-DM who engage in such behaviors require special attention in health services to improve their PT treatment outcomes and ensure adequate glycemic control.

In Brazil, HIV-TB coinfection accounts for more than 7,000 new TB cases annually [26]. The poor prognosis in this population is associated with diagnostic challenges and low adherence to both HAART and TB treatment [27,28]. In this study, the AR measures for DM were higher among HIV-negative patients and HIV-positive patients receiving HAART, indicating a significant role of DM in increasing the risk of death from PT in these subgroups. However, this association was not observed among HIV-positive individuals who were not receiving HAART or whose treatment status was unknown.

Seemingly divergent findings were reported by Villalva-Serra et al., who analyzed Brazilian surveillance data from 2014 to 2019. Although their study population partially overlapped with that of the present analysis, they concluded that DM was not associated with unfavorable TB treatment outcomes in TB-HIV coinfected individuals. The discrepancies between the two studies may be explained by methodological differences. Villalva-Serra et al. defined “unfavorable outcome” as a composite variable encompassing death, therapeutic failure, treatment adherence, and TB recurrence, while categorizing HIV status as a single, homogeneous variable [29]. In contrast, the present analysis focused exclusively on death as the outcome and stratified HIV-positive patients by HAART status. This stratification was essential to account for the substantial differences between HIV-positive individuals who were receiving antiretroviral therapy and those who were untreated or had unknown treatment status.

Regarding the estimated impact of DM on PT fatality rates across the subgroups analyzed, three distinct patterns were identified.

High ARDM% among groups with low biological and social vulnerability

In these populations - such as individuals with higher education levels and young adults (18-39 years) - the baseline risk of death from PT in the absence of DM was low but increased substantially when DM was present, accounting for more than half of the PT deaths among those with PT-DM. For these groups, DM should be recognized as an important modifier of PT prognosis, even when demographic characteristics suggest a low baseline risk of death. Screening PT patients for undiagnosed DM should be considered within the Brazilian health system, as recommended elsewhere [5], to identify such cases early.

Low ARDM% among groups with high baseline risks of death from PT

In populations such as individuals with no formal education, the elderly, or the homeless, DM had a limited additional effect on PT-related mortality. In these cases, social vulnerability appears to be the main determinant of poor outcomes, irrespective of DM status, and should be the focus of targeted public health interventions. It is noteworthy, however, that in populations where the prevalence of DM is high (e.g., among the elderly), even a small relative increase in risk may translate into a significant number of excess PT deaths at the population level.

High ARDM% among socially vulnerable groups with low baseline PT fatality rates

These included residents of peri-urban areas, recipients of the BCTP, and incarcerated individuals. In these groups, the relatively low PT fatality rates in the absence of DM may reflect increased health monitoring opportunities. For instance, participation in the BCTP may facilitate regular contact with healthcare services for household members, while incarcerated individuals are routinely screened for PT upon admission and receive directly observed therapy, including monthly clinical and microbiological follow-up until medical discharge [27]. Further cohort studies targeting these populations could help elucidate these findings.

This study has limitations inherent to its observational design, which relies on secondary data, and the limited availability of variables, such as data on nutritional status and glycemic control. Furthermore, the absence of estimates from probabilistic multivariable models, controlling for confounding factors, limits the interpretation of the AR measure, restricting it to a descriptive context (as stated in the objective of this study). Adding to eligible patients not included in this study (17%), the “transfer” outcome category may include in part individuals who were in fact lost to follow-up. Additionally, mortality data from SINAN may be underreported, and the use of self-reported DM could have introduced classification errors, likely underestimating the true prevalence of DM among patients with PT. However, these limitations are expected to be largely non-differential, making it unlikely that they significantly affected the comparability between PT cases with DM and those without.

## Conclusions

DM was significantly associated with increased fatality among patients with PT-DM in Brazil. The highest ARDM% values were observed in individuals with higher education, those aged 18-39 years, residents of peri-urban areas, BCTP beneficiaries, and incarcerated populations. These findings highlight the need for enhanced monitoring and targeted care for these groups, as they face a substantially increased risk of PT-related mortality associated with DM, even when possessing otherwise favorable sociodemographic characteristics. Clinical guidelines should incorporate stratified management strategies based on the presence of DM among PT patients.
